# Supplementary material for: The nucleosome acidic patch directly interacts with subunits of the Paf1 and FACT complexes and controls chromatin architecture in vivo
Source: Nucleic Acids Res. 2019 Jun 21;47(16):8410–23. doi: 10.1093/nar/gkz549 (PMC6895269; doi:10.1093/nar/gkz549)
Supplement: gkz549_Supplemental_File [file gkz549_supplemental_file.pdf]

Cucinotta et al. Fig. S1

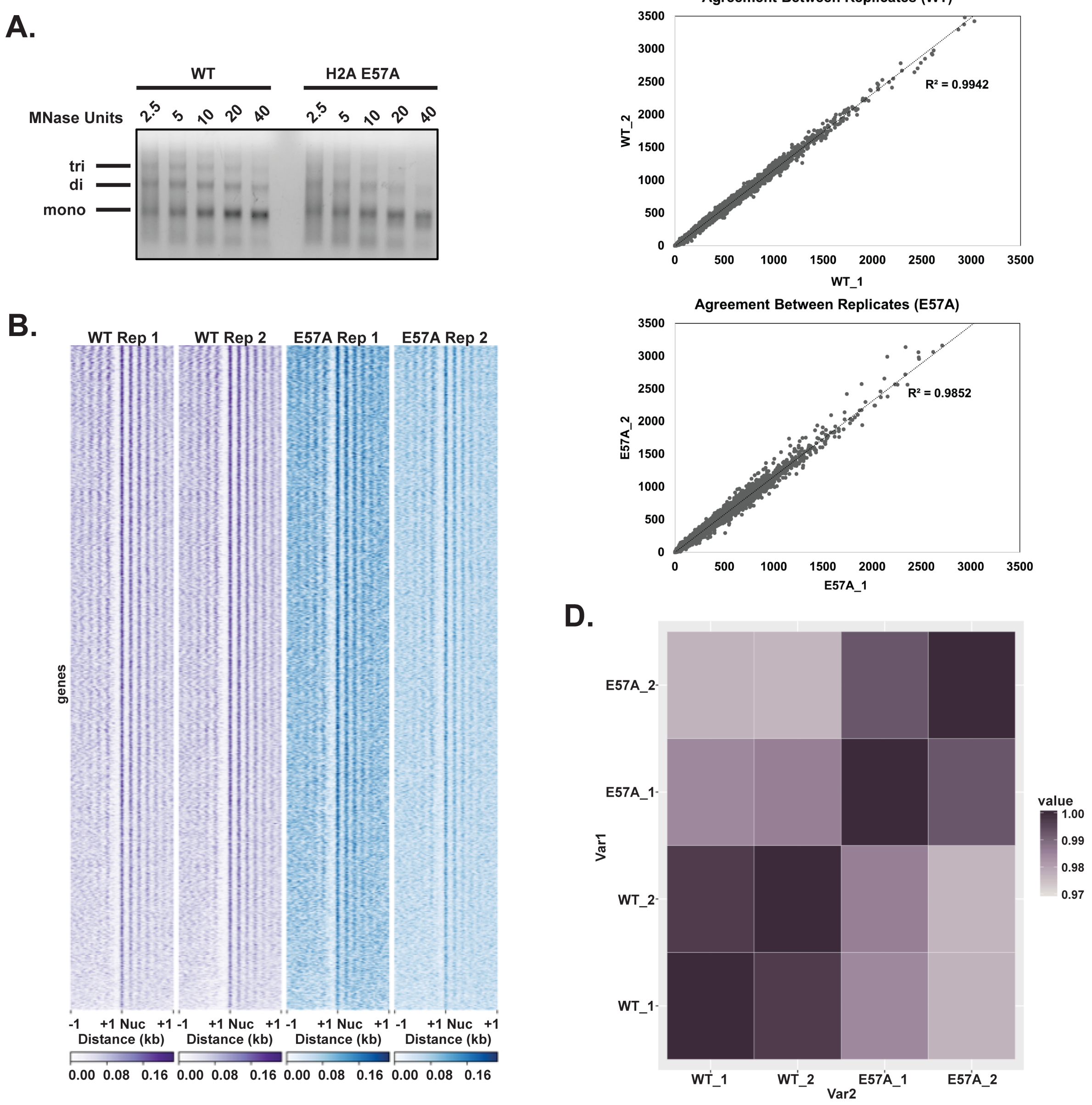

Figure S1. MNase titration of cell lysates and analysis of MNase-seq data reproducibility. **A.** Increasing units of MNase were added to cell lysates. Bottom band shows the mononucleosome species that was purified and subjected to library preparation and paired-end sequencing. **B.** Heatmaps of nucleosome positions determined in biological duplicate by MNase-seq analysis of strains expressing plasmid-encoded wild-type H2A or H2A-E57A as the only source of H2A. Sequences are aligned to the +1 nucleosome of annotated yeast genes. **C.** Biplots of MNase sequencing signal averaged over each annotated yeast gene comparing biological replicates of wild-type (top) or H2A E57A (bottom). R^2 values are indicated to the right of the line of best fit. **D.** Pearson correlation heat maps of MNase-seq data sets from all four samples. Darker color indicates higher correlation.

Cucinotta et al Fig. S2

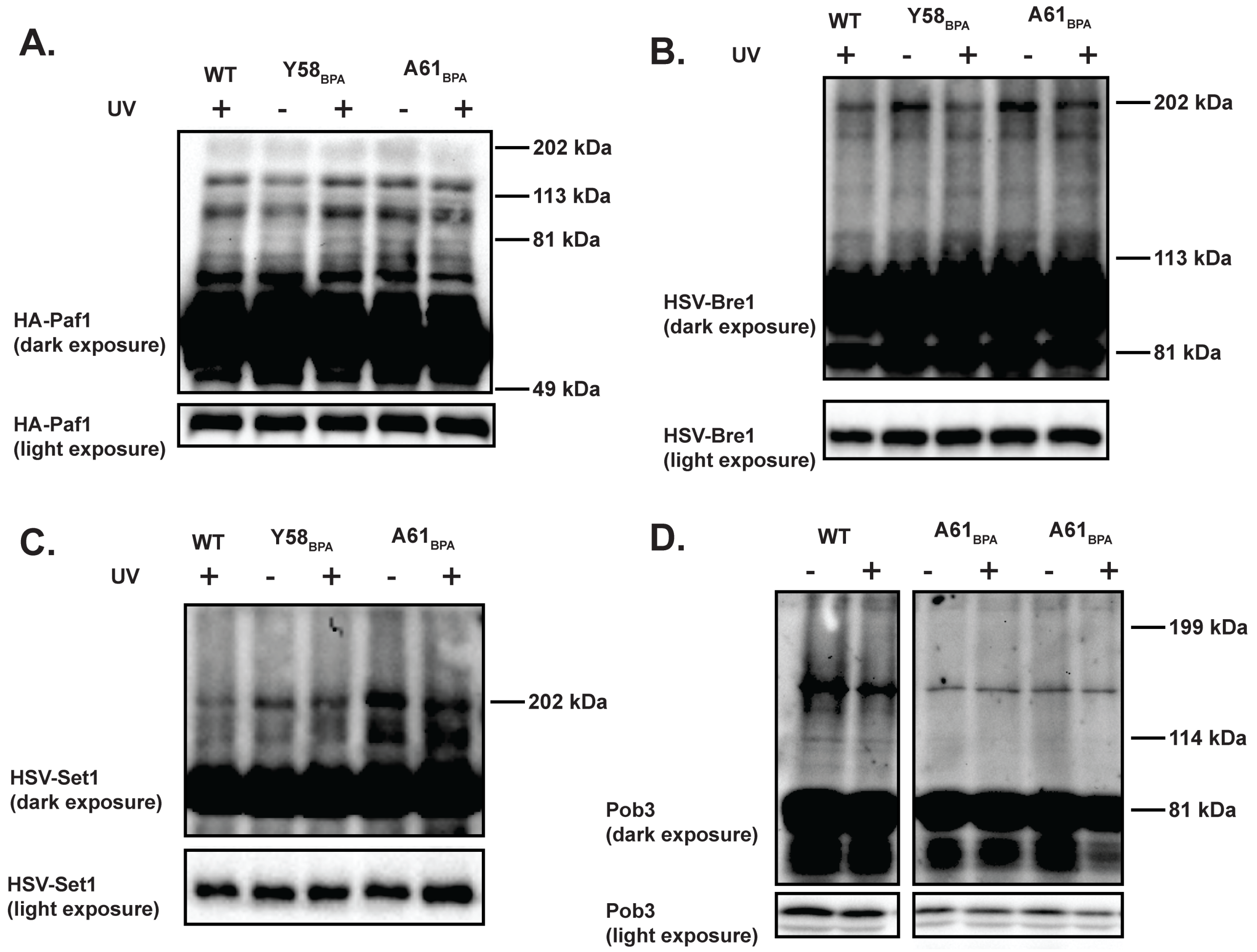

Figure S2. Proteins for which crosslinking to H2A-A61$_{BPA}$ was not detected
A-C. Western blot analysis of HA-Paf1 (A), HSV-Bre1 (B), and HSV-Set1 (C) in extracts prepared from a wild-type H2A control strain or strains expressing the H2A-Y58$_{BPA}$ and H2A-A61$_{BPA}$ derivatives. Cells were exposed to UV light as indicated. Top panel is a long exposure of the western blot and bottom panel is a short exposure of the same western blot. All three western blots are representative of biological duplicates. **D.** Western blot analysis of Pob3 in extracts prepared from a wild-type H2A control strain or a strain expressing the H2A-A61$_{BPA}$ derivative. Cells were exposed to UV light as indicated. The top and bottom panels are different exposures of the same western blot. The A61$_{BPA}$ experiment is shown in biological duplicate.

Cucinotta et al Fig. S3

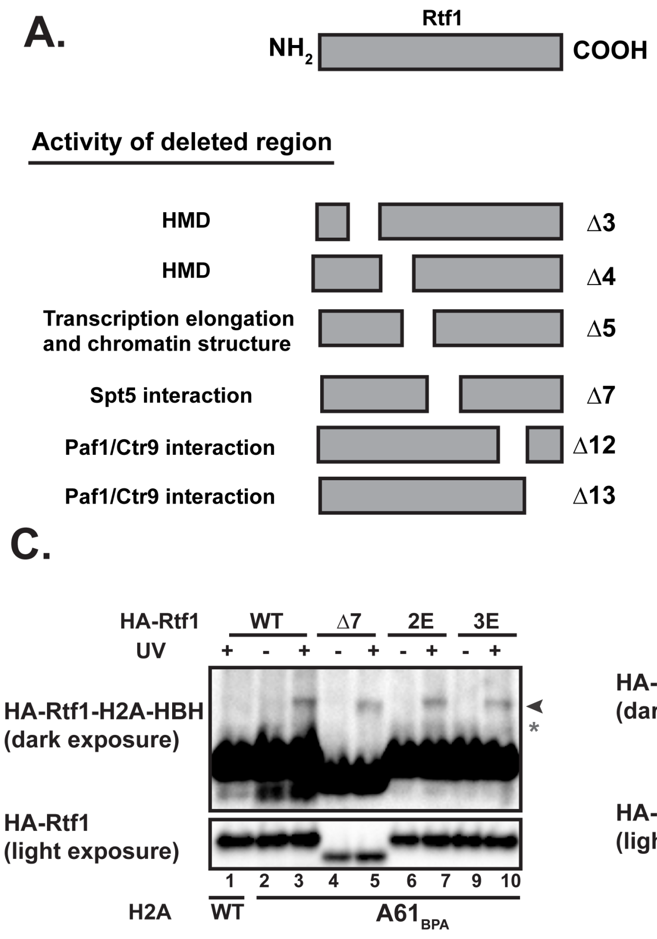

Figure S3. Domain mapping of the H2A-Rtf1 interaction
A. Diagram of Rtf1 deletion mutants and the function(s) of the corresponding domain that has been deleted (38). Western analysis of H2A-HBH crosslinking to wild-type HA-Rtf1 or HA-Rtf1 derivatives lacking region 5 **(B)**, lacking region 7 or containing glutamic acid substitutions for amino acids R273 and R288 (2E) or R251, R273, and K299 (3E) in region 7 **(C)**, or lacking regions 12 and 13 **(D)**. Arrows indicate crosslinked species. Experiments involving deletion mutants were performed in biological triplicate and experiments involving substitution mutants were performed in biological duplicate. Asterisks indicate the presence of non-UV specific cross-reacting bands. Note that the Rtf1 deletion derivatives migrate faster than the full-length Rtf1 as expected.

Cucinotta et al Fig. S4

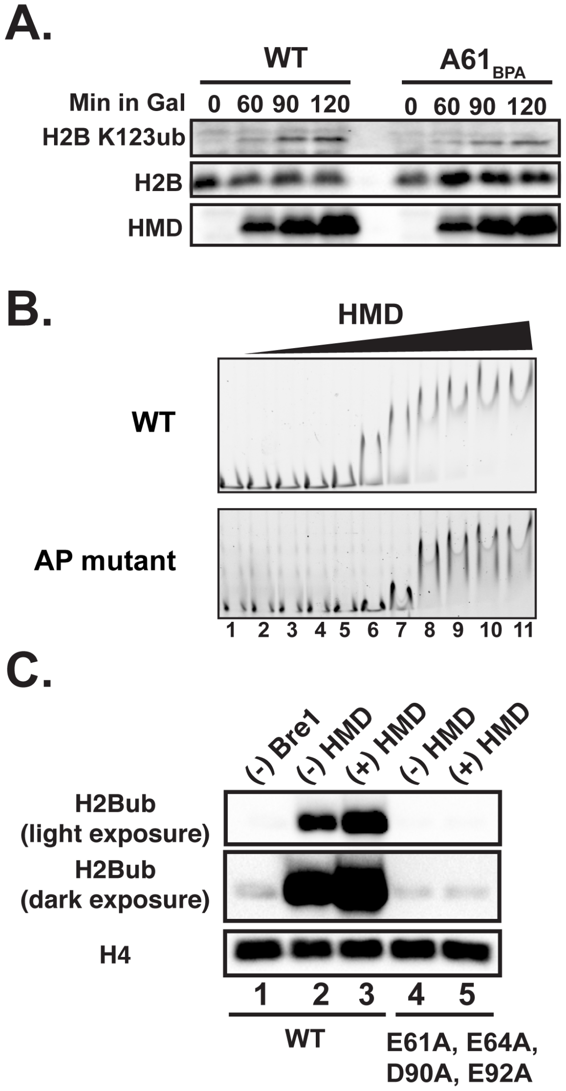

Figure S4. Analysis of HMD binding to the acidic patch
A. Time course of HMD induction in cells expressing the HMD under the control of the *GAL1* promoter. Western blots represent biological duplicate experiments, which were probed with antibodies against H2B K123ub, H2B, and Rtf1 (detects the HMD). **B**. EMSAs showing binding of purified, recombinant HMD_{74-184} to wild-type and mutant recombinant *X. laevis* nucleosomes. Lane 1 has no HMD present. The following amounts of HMD were used in lanes 2-11: 1 nM, 3 nM, 10 nM, 30 nM, 100 nM, 300 nM, 1 µM, 3 µM, 10 µM, and 30 µM. **C.** *In vitro* ubiquitylation assay, performed in duplicate, of wild-type and acidic patch mutant nucleosomes. Except where indicated, all ubiquitylation reactions contained recombinant E1 (UBE1), E2 (Rad6), E3 (Bre1) and ubiquitin. Purified HMD_{74-184} was added or omitted as indicated. The reaction in lane 1 lacked both Bre1 and HMD.

Cucinotta et al Fig. S5

A.

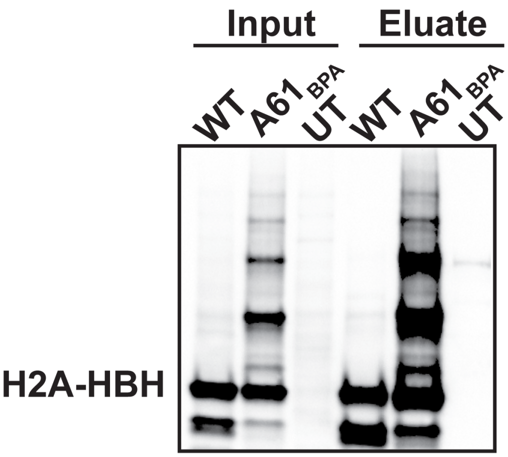

B.

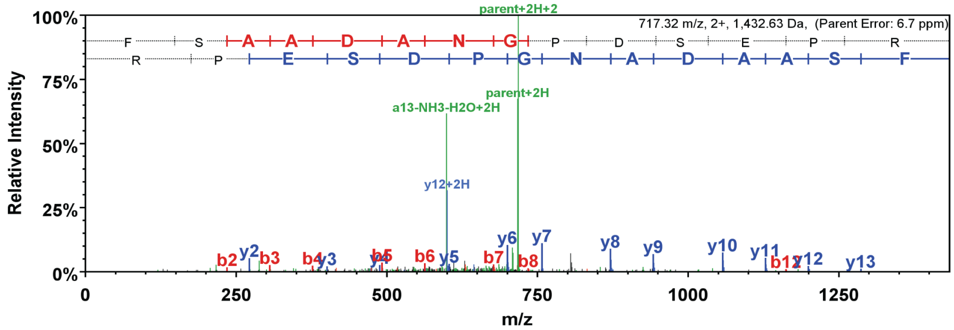

Figure S5. Mass spectra of Spt16 peptides detected after pulldown of H2A-A61$_{BPA}$-HBH from UV-exposed cells

A. Anti-his western blot analysis of eluates from nickel pulldown reactions performed in denaturing conditions for strains expressing H2A-HBH, H2A-A61$_{BPA}$-HBH, or an untagged H2A control (UT) and exposed to UV radiation. This experiment was performed in biological duplicate. **B**. Mass spectra of Spt16 peptides from one of the H2A-A61$_{BPA}$-HBH nickel pulldown experiments using extracts prepared from UV-treated cells under denaturing conditions.

Cucinotta et al Fig. S6

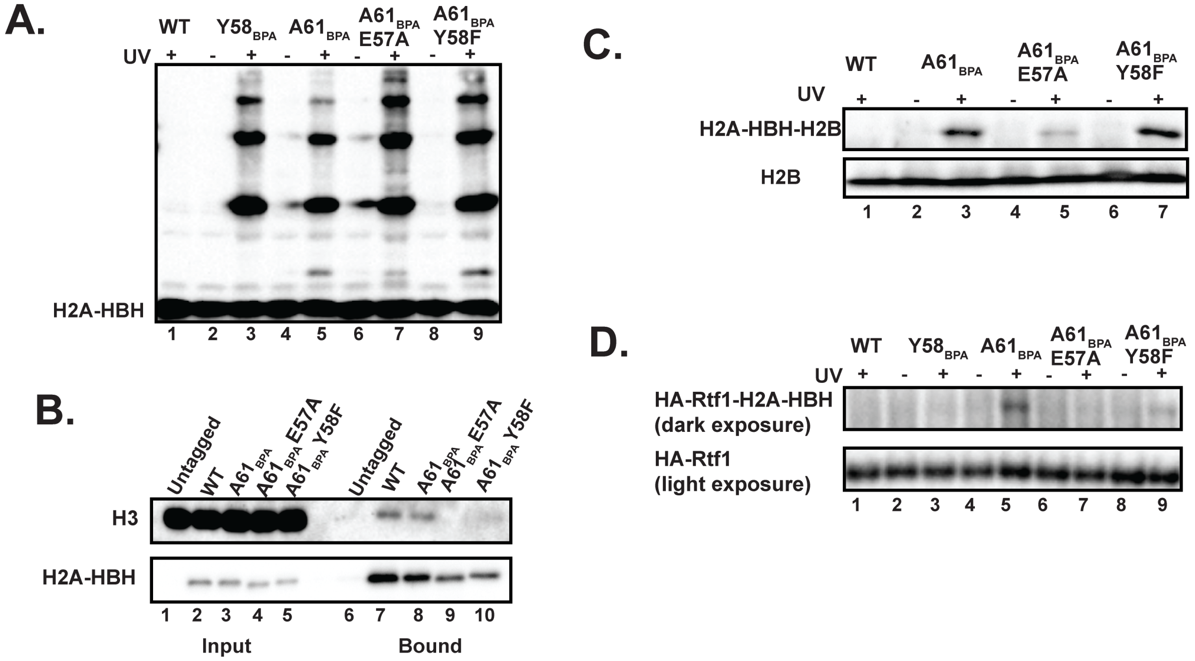

Figure S6. An intact acidic patch is required for H2A-A61$_{BPA}$ to interact with H3
A. Western blot analysis of extracts from UV-treated cells probed with anti-his antibody to detect the HBH tag appended to the indicated H2A derivatives. **B.** Western analysis of nickel pulldown experiments to analyze binding of the H2A-HBH derivatives to H3 under noncrosslinking conditions. Note that lanes 1-3 and 6-8 from these same blots are also shown in Figure 1D. **C.** Western blot analysis of H2A-H2B crosslinking from whole cell lysates. Top panel represents cross-linked species. Bottom panel is the non-crosslinked total H2B. **D.** Western blot analysis of H2A-HBH crosslinking with HA-Rtf1 in the context of H2A double mutants.

Supplementary Table S1. Yeast Strains

Strain	*MAT*	Genotype
KY2808	α	*his3Δ200 lys2-128δ leu2Δ1 ura3-52 3XHA-RTF1*
KY860	a	*his3Δ200 lys2-128δ leu2Δ0 ura3Δ0*
KY2788	α	*his4-912δ lys2-128δ ura3-52 trp1Δ63 3XHA-RTF1 bre1Δ::kanmx*
KY3047	a	*his3Δ200 lys2-128δ leu2Δ1 ura3-52 3XHA-RTF1 rad6Δ::kanmx*
KY680	α	*his4-912δ lys2-173R2 leu2Δ1 ura3-52 trp1Δ63 3XHA-rtf1Δ1*
KY2032	a	*his4-912δ lys2-128δ leu2Δ1 trp1Δ63 ura3-52 3XHA-rtf1Δ3*
KY2033	a	*his4-912δ lys2-128δ leu2Δ1 trp1Δ63 ura3-52 3XHA-rtf1Δ4*
KY1155	a	*his3Δ200 leu2Δ1 ura3-52 trp1Δ63 3XHA- rtf1Δ5*
KY1157	a	*his3Δ200 leu2Δ1 ura3-52 trp1Δ63 3XHA- rtf1Δ7*
KY2423	a	*his4-912δ lys2-128δ leu2Δ1 ura3-52 trp1Δ63 3XHA-rtf1-R251E-R273E-K299E*
KY2424	a	*his4-912δ lys2-128δ leu2Δ1 ura3-52 trp1Δ63 3XHA-rtf1 -R273E-R288E*
KY1159	a	*his3Δ200 leu2Δ1 ura3-52 trp1Δ63 3XHA- rtf1Δ*12
KY1420	α	*his3Δ200 leu2Δ1 ura3-52 trp1Δ63 3XHA- rtf1Δ13*
KY977 (FY2365)	a	*his3Δ200 lys2-128δ leu2Δ1 ura3-52 SPT16-3XMYC*
KY3046	α	*his3Δ200 lys2-128δ leu2Δ1 ura3-52 3XHA-RTF1 sgf11Δ::KANMX*
KY943 (FY406)	a	*(hta1-htb1)Δ::LEU2(hta2-htb2)Δ::TRP1 his3Δ200 lys2-128δ leu2Δ1 ura3-52* [pSAB6 = *URA3/C/A/HTA1-HTB1*]
KY3139	α	*his4-912∂ leu2Δ1 trp1Δ63 rtf1::TRP1-GAL1p-NLS-MYC-HMD74-184::KanMX*
KY1221	a	*his3Δ200 leu2Δ1 ura3-52 trp1Δ63* 3XHA-*PAF1*
KY2697	α	*lys2-128δ his3Δ200 leu2Δ1 ura3-52 3XHSV-BRE1*
KY1642	*a*	*his3Δ200 leu2Δ1 trp1Δ63 arg4-12 3XHSV-SET1*

Supplementary Table S2. Oligonucleotides

Primer	Dir.	Sequence 5' → 3'	Ref.
Amplify *HTA1* 450 bp up and down ATG	F<br>R	ATCAGAGCTCGCGCTGTTCCAAAATTTTCGCC<br>ATCACTCGAGGCGTATATATATATACAAATATGCG	(54)
Back bone for gibson assembly to add HBH tag	F<br>R	TAAGATCGGTTCTGGTATTTTAAAG<br>TAATTCTTGAGAAGCCTTGG	This study
Insert for Gibson assembly to add HBH tag to H2A	F<br>R	AAGGCTTCTCAAGAATTATTAATTAACAGGGGTTCACATC<br>TACCAGAACCGATCTTAAGATCTATATTACCCTGTTATCC	This study
Y58TAG SDM	F<br>R	ACTTGACTGCTGTCTTGGAATAGTTGGCCGCTGAAATT<br>TCTAAAATTTCAGCGGCCAACTATTCCAAGACAGCAGTC	This study
A61TAG SDM	F<br>R	CTTGGAATATTTGGCCTAGGAAATTTTAGAATTAGC<br>CAGCTAATTCTAAAATTTCCTAGGCCAAATATTCCAAGAC	This study
Amplify HMD_{74-184} for pAP39 cloning	F<br>R	GCTATTCCATATGGAAGAAGAAGCTAATCCTTTTCCCTTG<br>GGGAATTCTCATCATTCATTATCGCTGTAGTGACGGTTTTTCC	This study
Amplify HMD_{74-184} for pFA6a-KanMX cloning	F<br><br>R	TGTAATTGTATTGCACTAATTTGTTGAGAGCACTATAGAAATGCCAAAGAAGAAGAGAAAGGTTGG<br>GCATGGCCTTGTTCCTTGGCACGAAACCATTCACATCACGAATTCGAGCTCGTTTAAAC	This study
Integrate *GAL1p* at HMD	F<br><br>R	TGTAATTGTATTGCACTAATTTGTTGAGAGCACTATAGAAGAATTCGAGCTCGTTTAAAC<br>GACTAGTTTGTGGAATACCAACCTTTCTCTTCTTCTTTGGCATTTTGAGATCCGGGTTTT	This study

Supplementary Table S3. Plasmids

Plasmid	Purpose	Derivation and reference
pCEC09/KB1473	Untagged H2A	This study; (54)
pCEC21/KB1474	WT H2A-HBH	Gibson assembly of pCEC09
pCEC28/KB1476	H2A-$Y58_{BPA}$-HBH	Site-directed mutagenesis of pCEC09
pCEC29/KB1475	H2A-$A61_{BPA}$-HBH	Site-directed mutagenesis of pCEC09
pCEC30/KB1477	H2A-$A61_{BPA}$-E57A-HBH	Site-directed mutagenesis of pCEC29
pCEC31/KB1478	H2A-$A61_{BPA}$-Y58F-HBH	Site-directed mutagenesis of pCEC29
pLH157/*LEU2*	tRNA/tRNA synthetase containing plasmid	(40)
pKB1463	Myc-NLS-Rtf1-HMD_{74-184}	This study
pKB1464	Myc-NLS-Rtf1-HMD_{74-184}-KanMX	This study
pAY01	Plasmid containing *HTA1* and *HTB1*	(11)
pCEC02	Plasmid containing *hta1-E57A* and *HTB1*	(11)
